# Is there any relationship between physical activity level and patterns, and physical performance in children?

**DOI:** 10.1186/1479-5868-8-122

**Published:** 2011-11-04

**Authors:** Aurélie Blaes, Georges Baquet, Claudine Fabre, Emmanuel Van Praagh, Serge Berthoin

**Affiliations:** 1 University of Lille Nord de France, UDSL, EA 4488, Physical Activity - Muscle - Health, Lille, France; 2 Blaise Pascal University, UFR STAPS, Laboratory of Exercise Biology (BAPS), EA 3533, Clermont-Ferrand, France

## Abstract

**Background:**

It is often assumed that physical activity (PA) and physical performance during childhood and adolescence are beneficial for health during adulthood, but a positive relationship between PA and physical performance has not been precisely clarified in children. The lack or the weakness of the relationships between PA and physical performance could be due to the measure of PA. If the use of accelerometry is considered as an objective and common measure of PA, the real patterns of children's habitual PA must be reflected. The aim of this study was to investigate the relationship between the levels and patterns of PA assessed with high frequency accelerometry and physical performance in young children.

**Methods:**

Eighty-six boys and 101 girls aged 6-12 years participated in this study. Physical activity was measured over a 7-day period, using a 5-s epoch. Physical performance was assessed by means of EUROFIT tests (anthropometrics, standing broad jump, the 10 × 5 meter shuttle run, the sit-and-reach, the handgrip, the number of sit-ups in 30 seconds, the 20-meter shuttle run).

**Results:**

No relationship was found between PA and physical performance. In boys only, body fatness was negatively associated with vigorous PA (r = -0.38, p < 0.001) and very high PA (r = -0.35, p < 0.01), in contrast to light PA (r = 0.28, p < 0.01), which was positively related to body fatness.

**Conclusion:**

In 6- to- 12 year- old children, the more active children were not the fittest. Our results also underline the need for uniformity in approach to measurement of PA, body composition and health-related fitness between studies.

## Background

It is well established that physical activity (PA) is an important health determinant in young people. Sirard and Pate [1] reported an inverse relationship between PA and chronic diseases such as obesity, cancer, ischemia and coronary disease. Active children appear to be engaged in a sufficient variety of activities that enhance multiple components of health-related fitness [2]. The literature shows that a physically active lifestyle during childhood and adolescence may decrease the risk of having health problems during adulthood and that more active children tend to be more active adults [3,4]. Thus, it is often assumed that PA and physical performance during childhood and adolescence are beneficial for health during adulthood [5], but a positive relationship between PA and physical performance has not been precisely clarified in children [6,7]. Although Katzmarzyk et al. [8] have reported a significant relationship between PA and health-related physical performance, they noticed that a large part of the variability (80-90%) in performance is not accounted for by PA. The dose-response relationship between physical activity and performance and health during childhood remains incomplete and is not fully understood [9]. The lack or the weakness of the relationships between PA and physical performance could be due to the measure of PA. From an observational study, Bailey et al. [10] have provided objective evidence on the highly transitory nature of children's PA. Thus questionnaires or monitoring children's PA with a 1-min sampling do not reflect the real patterns of children's physical activity. The use of accelerometry is considered as an objective (frequency, intensity and duration) and common measure of children's habitual PA [11]. Using such a device with a 2-s epoch, Baquet et al. [12] demonstrated that the mean PA bout duration lasted about 20s and that 95% of high intensity activities lasted less than 10s with a median duration of 4s. Moreover, if vigorous and very high intensity activity bouts represented 2.4% of the daily PA, they accounted for 36.1 ± 5.8% of the amount of the total daily PA. Thus, monitoring with a 1-min epoch does not discriminate between high and very high intensity PA that can be related to physical performance.

Thus, to assess in detail the PA patterns in children, a high frequency monitoring is needed [12,13]. These studies have reported the nature of children's spontaneous activity patterns, but not their impact on health outcomes and physical performance. In adults, Murphy et al. [14] reported that, at least for aerobic fitness, accumulated and continuous patterns of exercise training of the same total duration confer similar benefits. In boys, Stone et al. [15] showed that the frequency of short bouts of PA were strongly related with health than longer bouts and reported a significant relationship between PA patterns and peak of oxygen uptake. However, to our knowledge, it is not known if PA patterns (frequency, intensity and duration) are related to other physical performance in boys and in girls. Some physical performances (speed, explosive strength) could be related with PA bout frequency and intensity and other (aerobic fitness) with PA bout duration. Therefore, the purpose of the present study was to investigate the relationship between PA level (time spent in different PA intensity), PA patterns (number of bouts of PA according to their duration and intensity) and physical performance level in 6- to 12- year- old children, through high frequency accelerometry monitoring.

## Methods

### Participants

At the beginning, two hundred and fourteen children (98 boys and 116 girls), aged 6 to 12, participated in this study. The children were taken from 16 elementary classes in the North of France. Full advice about possible risks and discomfort with the protocol was given to the children and their parents. All children gave their assent to take part in the study and all parents signed a written informed consent. The study was designed in accordance with the ethical standards of the Helsinki Declaration of 2008, and received approval of the local "Consultative Committee for the Protection of Persons in Biomedical Research".

### Anthropometry

Height was measured to the nearest 0.1 cm with a wall stadiometer (Vivioz medical, Paris, France). Body mass was measured to the nearest 0.1 kg with a calibrated electronic balance (Tanita TBF 543, Tanita Inco, Iokyo, Japan). Waist and hip circumferences were measured with the subjects in a standing position with a non-elastic measuring tape. Waist circumference was measured at the midpoint between the lower border of the rib cage and the iliac crest, and hip circumference was measured at the widest part of the hip region. The waist/hip (W/H) ratio was then calculated. Percentage of body fat was estimated from skinfold (SF) thickness (0.1 mm). Measurements were assessed with a Harpenden caliper (Harpenden Inc.) at two sites (Triceps, and Calf). According to Slaughter et al. [16], the equations were: Estimated percentage of body fat = 0.735 (∑SF) + 1.0 and percentage of body fat = 0.61 (∑SF) + 5.0, for boys and girls, respectively.

### Physical Performance Assessment

Prior to participating in the study, the children were fully familiarized with the testing procedures. The children performed seven field tests from the European Physical Fitness (EUROFIT) test battery. According to EUROFIT test battery recommendations [17], anthropometrics (hip and waist circumferences, percentage of body fat, height and weight) were included in the EUROFIT items. This battery was developed between 1976 and 1986 due to an initiative of the European Council. The goals of this battery were to establish standardized tests in Europe to help teachers to assess the physical performance of their pupils in schools and to help in measuring the health-related performance of the population. To comply with EUROFIT standards, the recommendations of the Committee of Experts on Sports Research [17] were followed. Mahoney and Boreham [18] reported that the EUROFIT tests were reproducible in primary schools (113 children, 7 to 11 yrs-old, with a one month-interval assessment). The tests were the standing broad jump (SBJ, explosive strength, in cm), the 10 × 5 meter shuttle run (SHR, speed and agility, in s), the sit-and-reach (SAR, flexibility, in cm), the handgrip (HG, static strength, in kgf), the number of sit-ups in 30 seconds (SUP, abdominal muscular power), the 20-meter shuttle run (20-MST, maximal aerobic power, in km.h^-1^). Sixty randomly selected children (30 boys and 30 girls) performed the 20-m shuttle run test with continuous heart rate (HR) monitoring (Polar Accurex+, Finland) to assess the maximality of the test (Table 1). A HR value above 195 bpm was accepted as a maximal index, associated with visible exhaustion [19].

**Table 1:** Mean ± SD for anthropometric measurements and EUROFIT performances in boys and girls

	Boys (n = 86)	Girls (n = 101)
Age (yr)	9.1 ± 1.3	9.1 ± 1.3
Height (cm)	135.9 ± 9.5	135.7 ± 10.3
Body mass (kg)	33.3 ± 11.0	32.0 ± 8.0
Body mass index (kg.m^-2^)	17.6 ± 3.4	17.1 ± 2.5
Hip/waist circumference ratio	0.83 ± 0.06	0.81 ± 0.05
% BF (%)	15.1 ± 5.5	18.2 ± 4,1***
SBJ (cm)	129.2 ± 22.3	128.9 ± 23.6
SHR (s)	21.5 ± 2.6	21.9 ± 3.9
SAR (cm)	15.0 ± 6.1	18.3 ± 6.4***
HG (kgf)	15.6 ± 4.5	15.2 ± 4.0
SUP (n)	14.7 ± 4.9	14.9 ± 4.3
20-MST (km.h^-1^)	9.7 ± 0.8	9.7 ± 0.7
	Boys (n = 30)	Girls (n = 30)
Maximal HR during 20-MST (bpm)	200 ± 9	201 ± 12

% BF: percentage of body fat; SBJ: standing broad jump; SHR: 10 × 5 m shuttle run; SAR: sit and reach; HG: handgrip; SUP: number of Sit-ups; 20-MST: 20 meter shuttle run. Maximal HR during 20-MST: maximal heart rate during the 20 m shuttle run test.

***: significantly different between genders at p < 0.001.

As recommended by the EUROFIT guidelines, the subjects performed the tests in the following order: SBJ, SHR, SAR, HG, SUP and 20-MST. Each test was separated by at least 15-min. Testing was completed by all participants over one week.

### Physical Activity Assessment

Children's physical activity was assessed using an uniaxial accelerometer, over a 7-day consecutive period (The Actigraph, Manufacturing Technologies, Inc., model 7164, Fort Walton Beach, FL). Only the children who completed at least a 6-day monitoring were included for further analysis. The Actigraph accelerometer facilitates the quantification of human motion (frequency and intensity) over a user-specified time interval called an epoch. The acceleration signal is digitized and the magnitude is summed over the epoch. At the end of each epoch duration, the summed value or activity "count" is stored in memory, and the integrator is reset. For this study, the epoch duration was set at 5-s, and data between 7 am and 9 pm were retained to subsequent analysis. The Actigraph used in this study has been shown to be a valid and reliable tool for quantifying PA in children [20].

To maximize the quality of the data, strategies were employed to encourage children compliance. Children received oral and written information to use the accelerometers comfortably: they wore the accelerometers on the right hip fastened with an elastic belt from waking up until bedtime. The accelerometer was removed during bathing and swimming. The investigators demonstrated to the parents and children, how to wear the device properly and reminded parents and children of the importance of not forgetting to wear it.

All the files were visually inspected to delete missing data. If the child had forgotten the device at any time of the day (morning, afternoon and evening), the file was rejected. A sequence of 120 zero counts (10 min recording) was defined as the level of missing data [21]. The 5-s activity counts were uploaded to an Excel^© ^macro to calculate the time spent below and above different PA thresholds, corresponding to light (LPA < 3 METs), moderate (MPA, 3 ≤ MPA < 6 METs), vigorous (VPA, 6 ≤ VPA < 9 METs) and very high PA (VHPA ≥ 9 METs). The Actigraph outputs of 162, 440 and 790 counts per 5s were the cut-offs used to define 3, 6 and 9 METs, respectively [22]. Times spent below and above the different intensity thresholds were calculated for each of the 7 days. To examine continuous PA behavior patterns, the daily number of PA bouts of various durations [from 5 to 15s], [from 16 to 30s], [31 to 60s], [61 to 180s], [181 to 300s], [301 to 600s] and [> 600s] were calculated for each intensity level [12].

According to Strong et al. [7], the percentage of children who reached the PA international guideline (60 accumulated minutes from MPA to VHPA, each day a week) was calculated.

### Statistical analysis

The normality distribution of the data was checked using the Kolmogorov-Smirnov test. The experimental values were expressed as mean ± standard deviation (mean ± SD). A Student's *t-*test was used to identify differences between boys and girls in anthropometric data, times spent below and above the different intensity thresholds over time (one week); number of bouts according to their duration and intensity and the EUROFIT performances.

We hypothesized that physical activity would be significantly associated with physical fitness dimensions as assessed from physical performances. Some of these performances (speed, explosive strength) may be related with PA bout frequency and intensity and aerobic performance with PA bout duration. Univariate analyses were conducted to examine the relationships between PA, times spent at various PA intensities, and physical performance, using Pearson product moment correlations, adjusted for age and percentage of body fat. A multivariate stepwise regression analysis was developed a priori to account for variation for each physical performance with a correlation significant at a 0.2 p-level. Other PA variables were added to the basic model to determine which model explained the most variance with the fewest number of covariates. When significant relationships were observed between times spent at various PA intensities level and physical performance, relationships with the corresponding activity pattern variables were explored.

Statistical analyses were conducted with Instat 3 (GraphPad software). The threshold for statistical significance was set at p ≤ 0.05.

## Results

Although investigators and parents reminded children of the importance of wearing their device during the experimental period and tried to complete the measurement of physical performance, twenty-seven children (12 boys and 15 girls) were rejected for an incomplete PA data set or for absences in measured physical performances. One hundred and eighty-seven children (101 girls and 86 boys) were finally retained for the study.

### Anthropometry and physical performance

Age, anthropometric data and physical performance are presented in Table 1. The Student's *t*-test revealed no sex difference, except for a higher percentage of body fat and a higher flexibility in girls (p < 0.001).

### Physical activity

For boys and girls, the daily PA time spent at different intensity levels is presented in Table 2. Girls spent more time in LPA than boys (+17.4 min, *p *< 0.001), whilst the latter spent more time in MPA (+12.6 min, p < 0.001) and in VPA (+3.5 min, *p *< 0.01). No sex-related difference was found for time spent in VHPA. Boys (95.3%) were more likely to attain (p < 0.05) the current PA guidelines (i.e. accumulating at least 60 min of moderate to vigorous PA per day) than the girls (77.5%).

**Table 2:** Mean ± SD for daily times spent at various PA intensity levels between 7 am to 9 pm and percentage of boys and girls who reached the international PA guideline [7]

	LPA (min)	MPA (min)	VPA (min)	VHPA (min)	≥60 min MPA to VHPA (%)
Boys (n = 86)	745.6 ± 24.4***	69.5 ± 16.6***	16.3 ± 6.4**	7.6 ± 3.9	95.3
Girls (n = 101)	763.0 ± 21.1	56.9 ± 14.2	12.8 ± 5.2	6.5 ± 5.4	77.5

LPA: light physical activity; MPA: moderate physical activity; VPA: vigorous physical activity; VHPA: very high physical activity. ≥60 min MPA to VHPA (%): percentage of children which accumulated more than 60 min MPA to VHPA.

**: significantly different between genders at p < 0.01; ***: significantly different between genders at p < 0.001.

For the whole population, the mean duration of PA bouts was 95.4 ± 22.2s for LPA, 13.6 ± 2.8s for MPA, 8.3 ± 0.9s for VPA and 6.9 ± 0.7s for VHPA. Girls showed (p < 0.001) longer LPA bouts (100.3 ± 1.6s) than, the boys (89.5 ± 20.8s), while boys showed (p < 0.001) longer MPA bouts (14.8 ± 2.6s) than the girls (12.7 ± 1.6s). No gender difference was found for VPA and VHPA mean duration bouts.

Table 3 displayed the distribution of PA bouts according to their intensity and duration. As intensity increased from LPA to VHPA, there was a steady decrease in the frequency of bouts as a function of their respective durations. Girls showed significantly more 1- to 10-min LPA bouts than the boys (p < 0.01). Boys presented significantly more 16-s to 3-min MPA bouts (p < 0.001), more VPA bouts inferior to 30-s (p < 0.05) and more VHPA bouts shorter than 15-s (p < 0.001) than the girls.

**Table 3:** Mean ± SD for the daily number of continuous bouts of physical activity according to their duration and intensity

	LPA		MPA		VPA		VHPA	
	**Boys**	**Girls**	**Boys**	**Girls**	**Boys**	**Girls**	**Boys**	**Girls**

≤15s	214.4 ± 63.9***	174.3 ± 55.6	344.8 ± 79.1	336.3 ± 76.7	157.9 ± 53.4***	125.3 ± 48.4	59.9 ± 25.8***	44.1 ± 27.9
15 < x ≤ 30s	63.1 ± 16.3	60.5 ± 17.0	49.8 ± 14.9***	38.7 ± 12.9	9.4 ± 5.2***	7.0 ± 5.5	1.8 ± 1.7	1.8 ± 2.2
30 < x ≤ 60s	51.5 ± 12.6	53.9 ± 12.5	22.3 ± 8.5***	14.5 ± 6.3	1.9 ± 1.3	1.8 ± 1.8	0.3 ± 0.5	0.5 ± 0.9
60 < x ≤ 180s	52.1 ± 11.5	58.9 ± 11.0***	7.3 ± 3.4***	4.3 ± 2.5	0.07 ± 0.1	0.1 ± 0.3	0.1 ± 0.2	0.2 ± 0.4
180 < x ≤ 600s	27.6 ± 5.8	31.2 ± 5.1***	0.5 ± 0.5	0.5 ± 0.6	0.02 ± 0.08	0.003 ± 0.02	0.004 ± 0.02	0.03 ± 0.1
>600s	14.6 ± 2.9	14.6 ± 2.5	0.02 ± 0.7	0.02 ± 0.01	0.002 ± 0.02	0.005 ± 0.05		

LPA: light physical activity; MPA: moderate physical activity; VPA: vigorous physical activity; VHPA: very high physical activity.

***: significantly different between genders at p < 0.001.

### Relation between physical activity and EUROFIT tests

Relationships between physical performance and times spent at PA intensity levels are presented in Table 4. For girls, no aspect of physical performance was related to PA. In boys, percentage of body fat was positively correlated with LPA (r = 0.28, p < 0.01) and negatively with VPA and VHPA (r = -0.38, p < 0.001 and r = -0.35, p < 0.01, respectively). No relationship was found between time spent in MPA and physical performance.

**Table 4:** Correlations between the times spent at various intensity levels, anthropometry and EUROFIT performances for the boys and the girls.

Boys	Height (cm)	Weight (kg)	BMI (kg.m^-2^)	H/W	% BF	SBJ (cm)	SHR (s)	SAR (cm)	HG (kgf)	SUP (n)	20-MST (km.h^-1^)
LPA	-0.06	0.06	-0.11	-0.04	0.28**	-0.11	0.18	-0.07	-0.08	-0.15	-0.08
MPA	0.08	-0.02	-0.06	0.04	-0.19	0.06	-0.15	0.02	0.11	0.11	0.08
VPA	0.04	-0.09	-0.15	0.05	-0.38***	0.18	-0.18	0.12	0.03	0.20	0.08
VHPA	0.06	-0.06	-0.12	-0.02	-0.35**	0.18	-0.19	0.08	-0.004	0.21	0.06

Girls											

LPA	-0.05	-0.08	-0.08	-0.06	0.03	0.10	0.05	-0.04	-0.03	-0.12†	-0.10
MPA	0.02	0.06	0.08	0.09	-0.01	-0.15	-0.02	0.07	0.02	0.12†	0.10
VPA	0.04	0.05	0.05	0.04	-0.04	-0.08	-0.07	-0.01	-0.001	0.11	0.16†
VHPA	-0.19†	-0.18†	0.12	-0.01	-0.15†	-0.16	0.08	-0.05	-0.14	-0.08	-0.01

LPA: light physical activity; MPA: moderate physical activity; VPA: vigorous physical activity; VHPA: very high physical activity. BMI: body mass index; H/W: hip/waist circumference ratio; % BF: percentage of body fat; SBJ: standing broad jump; SHR: 10 × 5m shuttle run; SAR: sit and reach; HG: handgrip; SUP: number of sit-ups; MS: maximal shuttle speed.

**: p < 0.01 significance; **: p < 0.001 significance; †: p < 0.2.

The multivariate stepwise regression analysis was developed to account for variation in percentage of body fat for boys, and, in SUP and 20-MST for girls. Only a significant relationship was found for body fatness in boys (r^2 ^= 0.20, p < 0.01). VPA makes a significant contribution to the model, while LPA and MPA provided redundant information. LPA was not included in the model (Table 5).

**Table 5:** Variables (times spent at various PA intensities) explaining the percentage of body fat in boys

	%BF	
***R^2 ^(SEE)***	***0.20 (5.09)***	

	***Estimates***	***P values***
Age	0.05 (-0.02 - 0.12)	0.172
MPA	0.02 (-0.05 - 0.09)	0.547
VPA	-0.29 (-0.52 - -0.05)	0.018
VHPA	-0.27 (-0.62 - 0.08)	0.13

%BF: percentage of body fat; MPA: moderate physical activity; VPA: vigorous physical activity; VHPA: very high physical activity; SEE: standard error of the estimate. Confidence intervals: 95% of estimates are displayed in brackets.

Relationships between PA patterns and physical performance are presented in Table 6. In boys, long bouts of LPA (>600s) were positively related to higher percentage of body fat (r = 0.34, p < 0.01), while negative correlations were found with short and medium bouts of LPA (5 to 180-s, from r = -0.31, p < 0.01 to r = -0.23, p < 0.05), VPA (5 to 60-s, from r = -0.44, p < 0.001 to r = -0.25, p < 0.05) and VHPA (5 to 60-s, from r = -0.39, p < 0.001 to r = -0.24, p < 0.05. A multivariate stepwise regression analysis was developed to account for variation in percentage of body fat for boys. A significant relationship was found for percentage of body fatness in boys (r^2 ^= 0.26, p < 0.001). Short bouts of VPA (from 5 to 15s) make a significant contribution to the model (Table 7).

**Table 6:** Correlations between the number of bouts according to their duration and intensity and percentage of body fat in boys.

		% BF		% BF		% BF
≤15s		-0.31**		-0.44***		-0.39***
15 < x ≤ 30s		-0.30*		-0.38*		-0.26*
30 < x ≤ 60s	LPA	-0.25*	VPA	-0.25*	VHPA	-0.24*
60 < x ≤ 180s		-0.23*		-0.02		-0.09
180 < x ≤ 600s		-0.10		-0.02		-0.04
>600s		0.34**				

LPA: light physical activity; VPA: vigorous physical activity; VHPA: very high physical activity; % body fat: percentage of body fat.

*: p < 0.05 significance; **: p < 0.01 significance; ***: p < 0.001 significance.

**Table 7:** Variables (number of bouts according to their duration and their intensity) explaining the percentage of body fat in boys

	% BF	
***R^2 ^(SEE)***	***0.26 (4.91)***	

	***Estimates***	***P values***
Age	0.03 (-0.05 - 0.11)	0.51
LPA ≤ 15s	0.02 (-0.02 - 0.05)	0.31
LPA > 600s	0.42 (-0.012 - 0.96)	0.13
VPA ≤ 15s	-0.05 (-0.08 - -0.14)	0.006
VHPA 30 < x ≤ 60s	-1.57 (-3.84 - 0.71)	0.17

LPA: light physical activity; VPA: vigorous physical activity; VHPA: very high physical activity; %BF: percentage of body fat; SEE: standard error of the estimate. Confidence intervals: 95% of estimates are displayed in brackets

## Discussion

This study investigated the relationship between physical performance and PA patterns in prepubertal children by means of EUROFIT tests and high frequency accelerometry measurement. The main finding is that the physical performance was not related to physical activity level in 6- to 12-yr-old children. Only a trivial negative relationship between PA level and body fatness was observed in boys with short bouts of VPA (from 5 to 15s) that made a significant contribution in percentage body fat variation as demonstrated by the multivariate analysis.

From childhood to adolescence, the literature has generally shown a weak to moderate relationship between PA and physical performance [23]. We hypothesized that high frequency accelerometry monitoring (5s epoch) should be able to provide a more accurate measurement of children PA behaviors, notably to capture short bouts of VPA and VHPA that are generally diluted in PA measurement when accelerometer epoch is set at 1 min. In the current study, no relationship was found between PA and physical performance. As observed in adults [24], these results suggests that habitual activity did not show adequate intensity, volume, and duration to induce positive changes in motor and functional capacities. Indeed, VPA or VHPA are generally considered as the upper boundaries of physical activity domains in the context of health related studies. The cut off used in the present study for VPA and VHPA were derived from counts values associated with velocities of 6.4 km.h^-1 ^(6 METs) and 9.7 km.h^-1^(9 METs), respectively. However, these intensities of exercise remain largely lower than those reached in the context of performance. For instance, in the present study, the running velocity associated with 20-MST performance (around 10 km.h^-1^) was certainly not sustained for sufficient long periods to expect an improvement in aerobic fitness. Similarly, a 9.7 km.h^-1 ^velocity (VHPA) remained two times lower than a sprint velocity in such a population that is around 20 km.h^-1^. Thus, it could be hypothesized that systematic training and not time spent in VPA or VHPA is needed to increase physical performances such as SBJ, SHR or 20-MST.

Only for boys, time spent in LPA was positively correlated with body fatness, whereas VPA and VHPA were negatively associated. Rowlands et al. [25] reported a negative relationship between fatness and PA in 8- to 10-yr-old boys and girls, whilst Dencker and Andersen [26] reported only low to moderate inverse relationships between moderate to vigorous PA and body fatness with a comparable population. Using accelerometry, Abbott and Davies [27] and Dencker et al. [28] found significant relationships between VPA and VHPA and body fatness, but no relationship was reported with MPA. These findings agree with the present study, where trivial relationships between VPA and VHPA and body fatness were also observed. Conversely, Ness et al. [29] reported higher correlations between moderate to vigorous PA and body fatness in boys of comparable ages. These differences could be explained by an acute measure of fat mass (dual x-ray emission absorptiometry) in the latter study, while skinfold measurement, as used in the present study, remains a less robust method. Nevertheless, the weak relationship found between LPA and percentage body fat in boys only, should also be carefully interpreted. The cut-offs used [22] in the present study do not allow to differentiate between LPA and sedentary activity, the latter being included in LPA. Body fatness in boys may thus be more related to sedentary activity than to LPA. Few recent studies proposed thresholds to discriminate between LPA and sedentary activity [30-32] but none of them used a 5s- epoch. It is possible that the 5-s epoch might not be appropriate to assess "true" sedentary activities. Indeed, this type of activity would better assessed with longer epochs such as 1-min rather than 5-s epoch. The latter could lead to an overestimation of sedentary activity. For example, during spontaneous PA, where children alternate short bouts of PA with short recovery periods, the recovery bout would be classified as sedentary activity, whereas it is not.

Relationships between waist circumference and body composition seem to be associated with cardiovascular disease risk and suggested that PA may have a beneficial effect, notably with respect to overweight [33,34]. In the present study, no relationship was found between hip and waist circumferences and times spent from LPA to VHPA. Using accelerometry, Hussey et al. [35] reported a significant negative correlation between waist circumference and time spent in VPA in boys, but not in girls. Similar conclusions were drawn by Ness et al. [29].

In the literature, positive relationships between PA and aerobic fitness have generally been observed in children [25,26]. However, correlations reported in the literature are moderate, therefore Dencker et al. [26] suggested that the aerobically fitter children were not obligatorily the more active. The results found in the current study showed that aerobic fitness was not associated with overall physical activity. Differences between experimental protocols (epoch length and/or field vs laboratory tests) may explain these discrepancies.

Children's PA is characterized by rapid changes from rest to PA including vigorous intensities. Investigating PA patterns related to health gives information about how to promote PA during childhood. To the best of our knowledge, only one study [15] has investigated the relationship between PA patterns and physical fitness. They reported that children's sporadic activity was negatively related to waist circumference and aerobic fitness. In the present study, short bouts of VPA in boys were positively related to lower body fatness, whereas LPA and sedentary long bouts were negatively associated. Our results show that PA intensity has a positive influence on the maintenance of a healthy body composition and suggest that children should be engaged in longer VPA sessions. As the majority of children's VPA and VHPA is accumulated via short intermittent bouts rather than long continuous bouts, intermittent activity seems to be efficient to promote healthy body composition and health-related fitness. However, there is an urgent need for more longitudinal studies on children in which PA, physical fitness (or performance), and health are measured repeatedly in the same individual over an extended period of time [36,37].

More than ninety-five percent of the boys and 77.5% of the girls accumulated 60 or more minutes of moderate to very high PA per day. These results are higher than reported on UK children [15], but lower compared with some American or European studies [21,38]. However, the proportion of children reaching PA guidelines should be interpreted with caution. The use of higher accelerometer thresholds to classify intensity or the use of a smaller epoch to monitor PA might explain these discrepancies between studies.

Times spent from MPA to VHPA were lower than reported in the literature. Girls spent more time in LPA than boys (2.1%, p < 0.001), whilst the latter spent more time in MPA (1.5%, p < 0.001) and in VPA (0.4%, *p *< 0.05). A sex-related difference was only found for 5 to 10-s VHPA bouts (p < 0.001). Hussey et al. [35] assessed PA during 4 days on Irish children aged 7 to 10. They observed that boys spent twice as much VPA and VHPA as girls (64.3 min vs 37 min each day, p < 0.001). Trost et al. [39] reported that boys spent about 120 min in MPA, whereas girls spent 100 min per day (p < 0.05). These differences may be explained by the use of different accelerometers [35,40]; different epochs [21,39]; number of the monitoring days [41] and different cut-offs [30]. In the present study a 5-s epoch was used to assess more accurately children's PA patterns in free-living conditions. Vigorous PA and VHPA are captured and not diluted in MPA or LPA. Using a 2-s epoch, Rowlands et al. [13] reported that differences in PA were largely due to the intensity of the most frequent bouts of activity and the frequency of the most intense bouts. McClain et al. [42] also reported that shorter epoch lengths should be used to minimize error among individual estimates.

## Conclusion

In 6- to- 12 yr- old children, the children's PA level is poorly related to the physical performance. Only a trivial relationship between vigorous physical activity and body fatness was observed. Our results also underline the need for uniformity in approach to measurement of PA, body composition and health-related fitness between studies.

## Competing interests

The authors declare that they have no competing interests.

## Authors' contributions

AB and GB were involved in acquisition, analysis, and interpretation of data, drafting and manuscript writing. SB was involved in analysis and interpretation of data, drafting and critically revising the manuscript. CF and EVP were involved in the conception and design of the paper and played a role in critically revising and editing the manuscript. All authors read and approved the final manuscript.
